# The Glucocorticoid Receptor NR3C1 in Testicular Peritubular Cells is Developmentally Regulated and Linked to the Smooth Muscle-Like Cellular Phenotype

**DOI:** 10.3390/jcm9040961

**Published:** 2020-03-31

**Authors:** Harald Welter, Carola Herrmann, Nils Dellweg, Annika Missel, Christiane Thanisch, Henryk F. Urbanski, Frank-Michael Köhn, J. Ullrich Schwarzer, Annette Müller-Taubenberger, Artur Mayerhofer

**Affiliations:** 1 Biomedical Center, Cell Biology, Anatomy III, Ludwig Maximilian University of Munich, 82152 Planegg-Martinsried, Germany; carola.herrmann@bmc.med.lmu.de (C.H.); n.dellweg@campus.lmu.de (N.D.); annika.missel@bmc.med.lmu.de (A.M.); amueller@bmc.med.lmu.de (A.M.-T.); 2 Ibidi GmbH, 82166 Gräfelfing, Germany; cthanisch@ibidi.de; 3 Division of Neuroscience, Oregon National Primate Research Center, Beaverton, OR 97006-248, USA; urbanski@ohsu.edu; 4 Andrologicum, 80331 Munich, Germany; info@andrologicum.com; 5 Andrology Center, 81241 Munich, Germany; info@andromuc.de

**Keywords:** human male fertility, human testis, dexamethasone, elastin, glucocorticoid, cytoskeleton

## Abstract

Whether glucocorticoids (GC) can directly affect human testicular functions is not well understood. A predominant site of GC receptor (GR; *NR3C1*) expression in the adult testis are peritubular smooth muscle-like cells, which express smooth muscle actin (ACTA2), contract and thereby are involved in sperm transport. In contrast to the adult, neither GR nor ACTA2, or elastin (ELN) were detected in the peritubular compartment before puberty in non-human primate testes. In isolated human testicular peritubular cells (HTPCs), activation of GR by dexamethasone (Dex) caused the translocation of GR to the nucleus and stimulated expression of *ACTA2* and *ELN*, without affecting the expression of collagens. Cytoskeletal ACTA2-rearrangements were observed and were associated with an increased ability to contract. Our results indicate post-pubertal testicular roles of GC in the maintenance of the contractile, smooth muscle-like phenotype of peritubular cells.

## 1. Introduction

The glucocorticoid receptor (GR; *NR3C1*, nuclear receptor subfamily 3 group C member 1) is present in the human testis [1], yet little is known about its significance for testicular function. However, it is well documented that levels of high glucocorticoid (GC) in patients with Cushing′s disease are associated with impaired testicular functions [2,3]. Whether the effects of adrenal-derived GCs on these patients are due to direct testicular effects and/or may act indirectly via a hypothalamic-pituitary inhibition, remains unclear.

A recent publication described the cellular localization of NR3C1 in the human testis [4]. It was detected in all peritubular (myoid) cells. Some Leydig cells were also positive for *NR3C1*, while other interstitial cells were negative. The study also reported the presence of these receptors in endothelial and smooth muscle cells of blood vessels, as well as in a subset of spermatogonial cells. In contrast, the expression of *NR3C1* in Sertoli cells was found to be weak or negative. This staining pattern is generally similar to the one depicted in the images in the Human Protein Atlas and the results are in line with gene expression data at the RNA level. In summary, these data indicate a prominent localization of GR in peritubular cells, which define the specific compartment around the seminiferous tubules in situ.

Peritubular cells are also cells of the human testis that can be isolated and examined in vitro [5,6,7]. As the in situ characteristics of peritubular cells are well maintained and characterized in vitro [7,8,9], studies in these human testicular peritubular cells (HTPCs), in conjunction with human testicular sections, provide a unique experimental window into the human testis.

In situ, these cells and extracellular matrix form the peritubular compartment of the seminiferous tubules. The cells express smooth muscle actin (ACTA2), calponin (CNN1), and other typical cytoskeletal markers, as well as a number of genes for specific extracellular matrix proteins [10]. Accordingly, for example, the extracellular matrix of the peritubular cell wall contains, among others, collagens and elastin (ELN; see images in the Human Protein Atlas; [11]). Due to their smooth muscle-like phenotype, they are able to contract and relax and thereby transport sperm [6,12]. Based on immunohistochemical studies of smooth muscle markers, this important feature is reported to be reduced, or even lost in male (idiopathic) infertility [13]. Also, the smooth muscle-like characteristics of peritubular cells develop only at puberty in primates [14], highlighting the importance for male fertility and implicating hormones in its regulation.

In the present study, we studied the expression of GR in the testes of man and in a non-human primate species. We also employed HTPCs as a cellular model system to explore consequences of GR activation in particular after application of dexamethasone (Dex), a frequently used synthetic glucocorticoid. We focused on the smooth muscle and extracellular markers and show that Dex regulates smooth muscle characteristics of HTPCs.

## 2. Experimental Section

### 2.1. Human and Monkey Samples

Testicular biopsies for HTPC isolation and for immunohistochemistry were obtained from men 36–55 years of age (in total *n* = 11) with obstructive azoospermia but normal spermatogenesis as described [5,9,13]. The study was approved by the local Ethics Committee (Technical University of Munich, Faculty of Medicine; project 491/18S-KK), and scientific use of the cells was permitted by written informed consent from all of the patients. The experiments were carried out in accordance with the relevant guidelines and regulations, and participants provided written consent.

Post-mortem testicular samples were obtained from rhesus monkeys (*Macaca mulatta*) through the Oregon National Primate Research Center (ONPRC) Tissue Distribution Program, and were identical to the ones used in previous postnatal developmental studies [15,16]. The study included seven immature animals (ages: 6, 7, 186, and 282 days) as well as three adults (ages: 18 and 27 years). Testes were fixed in Bouin’s solution, embedded in paraffin, and cut into 5-µm-thick slices for immunohistochemistry.

### 2.2. Cell Culture

HTPC lines (see Section 2.1) were cultured in Dulbecco’s Modified Eagle Medium high glucose (DMEM; Gibco, Paisley, UK) with 10% fetal calf serum (FCS; Capricorn Scientific, Ebsdorfergrund, Germany), and 1% penicillin/streptomycin (P/S; BioChrom, Berlin, Germany) at 37 °C, 5% CO_2_ and 95% humidity [5,17]. Cells from passages 8–12 were used. When confluence was reached, cells were starved for at least 24 h in serum-free medium to synchronize the cell cycle. Dexamethasone (Dex) and mifepristone (RU486; 4,9-estradien-17α-propynyl, 11β-[4-dimethylamino] phenyl-17β-OL-3-One, Sigma Aldrich, St. Louis, MO, USA) were dissolved in ethanol and then diluted in medium. Ethanol at a final concentration of 0.08% served as solvent control. Concentration of both Dex and RU486 at 1 µM was chosen based on our pilot experiments.

### 2.3. Determination of Cell Number, Cell Size and Viability

An automated cell counting device (CASY system, Schärfe Systems, Reutlingen, Germany) was used for calculating cell integrity, cell number, and cell diameter as previously described [17]. ATP has been accepted as a valid marker of cellular viability. We therefore determined the effect of 1 µM Dex on cellular ATP content of HTPCs (*n* = 3) for 24 h using the firefly luciferase assay, CellTiter-Glo^®^ Assay-kit (Promega, Mannheim, Germany) following the manufacturer’s protocol. The luminescence was measured in a luminometer (BMG Labtech, Ortenberg, Germany).

### 2.4. Isolation of RNA and Protein

Isolation of RNA and proteins from HTPCs of a total of 3–8 different donors was performed as previously described [8,18].

### 2.5. Reverse Transcription (RT-PCR) and Quantitative Real Time PCR (qPCR)

Complementary DNA (cDNA) was synthesized from 0.4 μg RNA using dN12 random primer followed by conventional PCR as described earlier [8]. qPCR measurements using the QuantiFast SYBR Green PCR Kit (Qiagen, Hilden, Germany) were conducted on the LightCycler 96^®^ System (Roche Diagnostics, Penzberg, Germany) as outlined before [8]. Samples were run in duplicate and analyzed using a ΔΔCT calculation method [19]. Primers (see Table S1) were designed by the 3web software spanning at least one intron when possible. Amplicons were verified by agarose gel electrophoresis with Midori Green Advance DNA stain (Nippon Genetics Europe, Düren, Germany) followed by sequence analysis (GATC, Konstanz, Germany).

### 2.6. Western Blotting

Western blot analysis was performed with HTPCs whole cell lysates as described [20]. Protein samples (10–15 µg) were separated via 10% SDS-PAGE and subjected to immunoblotting. Membranes were incubated overnight with the same anti-GR antibody (1:2500) as used for immunohistochemistry at 4 °C, followed by IRDye800 labeled secondary antibodies for 1 h. Membranes were scanned with the infrared-based Odyssey Imaging System (Li-Cor, Bad Homburg, Germany) and quantified using the Image Studio software. Results were normalized to β-actin (1:5000, anti-β-actin mouse monoclonal antibody A5441, Sigma-Aldrich, St. Louis, MO, USA) serving as a loading control.

### 2.7. Immunohistochemistry (IHC) and Immunocytochemistry (ICC)

Paraffin-embedded samples from patients with normal spermatogenesis were processed according to the avidin-biotin-peroxidase method [8]. HTPCs in culture were fixed with cold 4% paraformaldehyde for 15 min, and IF staining was performed with a fluorescence-tagged secondary antibody (1:800; goat α-rabbit Alexa-488, Life technologies, Carlsbad, CA, USA) as described with slight modifications [21]. For both techniques, an anti-NR3C1 (=GR) primary antibody (1:500 for IHC, 1:50 for ICC; affinity-purified, polyclonal rabbit anti-human NR3C1, HPA004248 Sigma Prestige Antibodies, St. Louis, MO, USA) was used, which recognizes both GRα and GRβ isoforms. In further IHC experiments, the following primary antibodies were used: anti-ACTA2 (1:200, mouse monoclonal anti-actin, Sigma A5228, St. Louis, MO, USA), Col1 (1:200, affinity-purified, polyclonal rabbit anti-Col1, R1038, Origene, Rockville, MD, USA), and affinity-purified, polyclonal rabbit anti-human ELN (1:200, A5228, Sigma Prestige Antibodies, St. Louis, MO, USA). Negative controls consisted of rabbit IgG (2 µg/mL; Millipore, Billerica, MA, USA) instead of the primary antibody, or of omission of the first antibody. After immunohistochemical staining, sections were analyzed using a Zeiss Axioplan microscope (Carl Zeiss Microscopy, Oberkochen, Germany) and digitalized with Progres Gryphax camera and software (Jenoptik, Jena, Germany).

### 2.8. Immunofluorescence Labeling of Filamentous Actin by Phalloidin

For immunolabeling, HTPC cells grown on round 12-mm glass coverslips, were treated with Dex as described under Cell culture (Section 2.2), and cultivated in parallel to untreated control cells. After 24 or 72 h, cells were fixed with 15% picric acid/2% paraformaldehyde in 10 mM PIPES, pH 6.0, for 15 min, and permeabilized with 0.1% Triton for 10 min, followed by washing and incubation in blocking buffer (PBS plus 2% bovine serum albumin) for 30 min at room temperature. For visualization of filamentous actin, cells were stained with Atto 488- or Atto 550-phalloidin (Sigma Aldrich, St. Louis, MO, USA), and DNA was visualized by staining with DAPI (4′,6-diamidino-2-phenylindole). Samples were embedded using Dako mounting medium (Agilent Technologies, Santa Clara, CA, USA).

Images were acquired with a Z1 Axio Observer microscope (Carl Zeiss Microscopy, Jena, Germany) equipped with 40×/1.3 NA and 63×/1.4 NA PlanApo oil immersion objectives, HXP 120C light source, and Axiocam 506 mono camera. Images were recorded using Zen acquisition software, and processed using the imaging processing package Fiji [22].

### 2.9. Cell Contractility Assay

This method was performed as described according to the manufacturer’s instructions (CytoSelect; Biotrend, Köln, Germany) using TM 24-Well Cell Contraction Assay Kit (Floating Matrix Model) with minor changes. In brief, collagen matrices containing 100,000 cells in 500 µL volume per well were seeded into a 24-well plate. After 1 h of polymerization at 37 °C, collagen gels were covered with serum-free medium and cultured with daily changes of medium for 48 h. Dex (1 µM) and solvent control (EtOH) were added in duplicates, and contraction of the free-floating collagen gels was monitored over 72 h. Images of gel matrices 72 h after treatment were taken and analyzed using Fiji [22]. Changes of gel size after Dex treatment were expressed as a percent and compared to solvent control wells. The assay was performed with cells from four different patients.

### 2.10. Electric Cell Impedance Sensing (ECIS) Measurements

Electrical impedance measurements were performed using an Electric Cell Impedance Sensing (ECIS) setup Z Theta (Applied BioPhysics, New York, NY, USA, supplied by ibidi, Gräfelfing, Germany) and 8W10E array electrode chambers (ibidi, Gräfelfing, Germany). Electrodes were stabilized by pre-incubation with serum-free medium for 72 h.

One × 10^5^ HTPCs were seeded into electrode chambers (total volume 400 µL per well), and maintained at 37 °C in an incubator. After 24 h, cells were starved for at least 2 h with serum-free medium and cultured with daily changes of medium for 72 h. Experiments were performed in parallel with HTPCs from two different donor cell lines and in duplicates for each measurement.

### 2.11. Statistical Analysis

Results derived from real-time PCR and ATP assay are expressed as mean + SEM. Student’s *t*-test was used to compare two groups (Dex effects on mRNA levels, cell number, cell diameter, ATP content). One-way analysis of variance and all pairwise multiple comparison procedures were applied to the blocking experiment using RU486. A probability value of *p* < 0.05 was considered significant, and of *p* < 0.01 highly significant. A boxplot presentation was chosen to depict numerical data of Dex effect on cell number and cell diameter, i.e., line shows the mean, boxes show 25th and 75th percentile, whiskers represent 5th to 95th percentile. Paired *t*-test was employed (Prism, GraphPad Software 4.0a, Inc., San Diego, CA, USA).

## 3. Results

### 3.1. GR Expression in Human Testicular Biopsies of Adult Men with Normal Spermatogenesis

**Figure 1:**
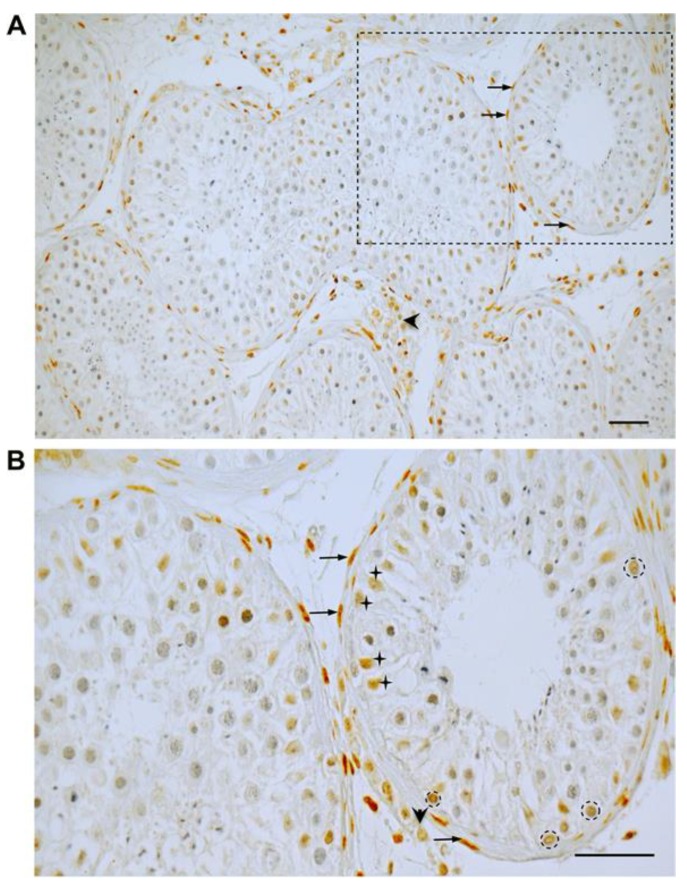
Immunohistochemistry of GR in the human testis (**A**,**B** = boxed area in A). In a representative human testicular biopsy, GR staining is found in peritubular, myoid cells (arrows in A,B) building the wall of seminiferous tubules, in some Sertoli cells (crosses, B) and some spermatogonia (circles, B). In addition, various interstitial cells, presumably Leydig cells (arrowheads in A,B), but also macrophages and endothelial cells of blood vessels, are immuno-positive. Nuclei were slightly stained with Hematoxylin. Scale bars = 50 µm.

Immunoreactive GR was located in the peritubular cells forming the wall of the seminiferous tubules (Figure 1A,B), some Sertoli cells and some spermatogonia (Figure 1B). Multiple GR-positive cells scattered in the interstitial compartment were also observed including presumably Leydig cells, macrophages, and endothelial cells of blood vessels. The staining results were identical in all four testicular samples examined. No labeling was detectable in control sections when the primary antibody was substituted by IgG (data not shown).

### 3.2. Age-Dependent GR Expression in the Testes of Rhesus Monkeys

**Figure 2:**
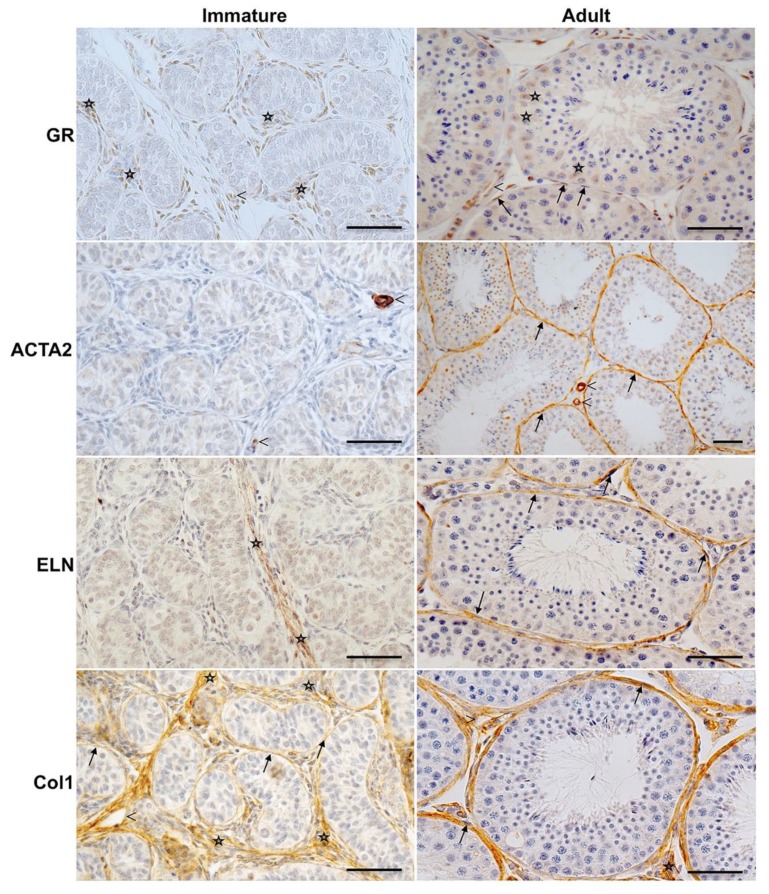
Expression of GR, ACTA2 and extracellular proteins in peritubular cells of the monkey testis before and after puberty. Immunohistochemistry of GR of adult rhesus monkeys (right panels) shows strong GR expression in the peritubular compartment (arrows) and moderate staining of Sertoli cells (asterisks). Smooth muscle cells of blood vessels (arrowheads) served as a positive intrinsic control. GR staining of the peritubular wall was not found in all samples from immature rhesus monkeys (186–282 days, left panels and results not shown). Only interstitial cells (asterisks) and blood vessels (arrowheads) show a robust staining. ACTA2 is constantly expressed by vascular smooth muscle cells (arrowheads) in both immature and adult monkeys but only localized in the peritubular wall of mature monkeys (arrows). In immature rhesus, staining of ELN is restricted to the interstitial matrix (asterisks) but is not detected in the peritubular compartment, which in turn is the prevailing expression site in adult monkeys (arrows). Independent of age, Col1 is localized in the peritubular compartment (arrows), in the interstitial area (asterisks), and around blood vessels (arrowheads). Negative control experiments were performed with IgG (not shown). Hematoxylin was used to counterstain nuclei. Scale bars = 50 µm.

As observed for the adult human testis, GR was expressed by peritubular cells surrounding the seminiferous tubules in the adult monkey (18–27 years) and was localized in vascular smooth muscle cells of testicular blood vessels (Figure 2). Some cells in the interstitial region were also GR-positive. GR expression in testicular samples of sexually immature rhesus monkeys was strikingly different. Generally, peritubular cells were not, or only occasionally, GR-immunoreactive. In detail, in testicular biopsies of newborn (6–7 days, data not shown) or immature monkeys (186–282 days), GR was solely expressed by vascular smooth muscle cells and in some cases found in interstitial cells (Figure 2). Similarly, ACTA2 was strongly expressed by vascular smooth muscle cells in both immature and adult monkeys, but was not detected in cells of the developing seminiferous peritubular wall of immature monkeys. Likewise, cells of the peritubular wall of adult monkeys stained immuno-positive for ELN, while it was not expressed in slices stemming from immature animals, except for some small interstitial areas. In contrast to ACTA2 and ELN, collagen 1 (Col1) was detectable around the seminiferous tubules and in interstitial tissue independently of age.

### 3.3. Expression and Induction of Dex-Dependent Translocation of GR Into the Nucleus

**Figure 3:**
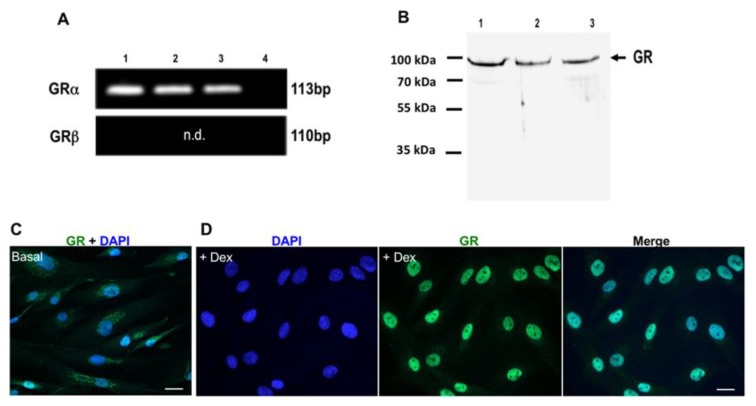
Expression of GR in human testicular peritubular cells (HTPCs). (**A**) Representative RT-PCR products from HTPCs of three different donors (1–3). A single band at the expected size of 113 bp is depicted, which upon sequencing was found to correspond to human GRα. Negative control (4) including RNA instead of cDNA shows no band. GRβ is not detected (n.d.) by RT-PCR. (**B**) Western blot of GR was performed with 10 µg of total protein extracted from three different HTPC patient lines (1–3), which reveal a band at 95 kDa. The membrane was stripped and re-probed for GAPDH (37 kDa) as loading control (not shown). (**C**) HTPCs grown in DMEM medium were fixed after 1 h, and the localization of GR was analyzed by immunofluorescence (Basal). Nuclear DNA was stained with DAPI. Under basal conditions, the GR is mainly localized in the cytoplasm, whereas the nuclei are nearly devoid of GR. (**D**) After Dex treatment (1 µM) for 1 h, GR is identified primarily in the nuclei, indicating translocation of GR after treatment. Scale bars = 10 µm.

To specify which subtype (α and/or β) of the GR receptor is expressed, analysis of HTPCs by RT-PCR was performed using primers designed to detect GRα and GRβ (Figure 3A). Results indicated the presence of a 113 bp transcript corresponding to GRα (verified by sequencing), while GRβ transcripts were not found. The use of a second GRβ primer set confirmed that this GR isoform is below detection level in HTPCs (data not shown).

Western blot analysis revealed the expression of the predicted full-length GR as a single band with an apparent molecular mass of approximately 95 kDa in HTPCs (Figure 3B).

As shown by immunocytochemistry (Figure 3C), in HTPCs exposed to medium (Basal = control), GR was mainly localized in the cytoplasm, whereas the nucleus remained nearly free. Upon exposure to Dex (1 µM; Figure 3D) for 1 h, a nuclear staining pattern of GR prevailed (merge), indicating rapid GR translocation after Dex binding.

**Figure 4:**
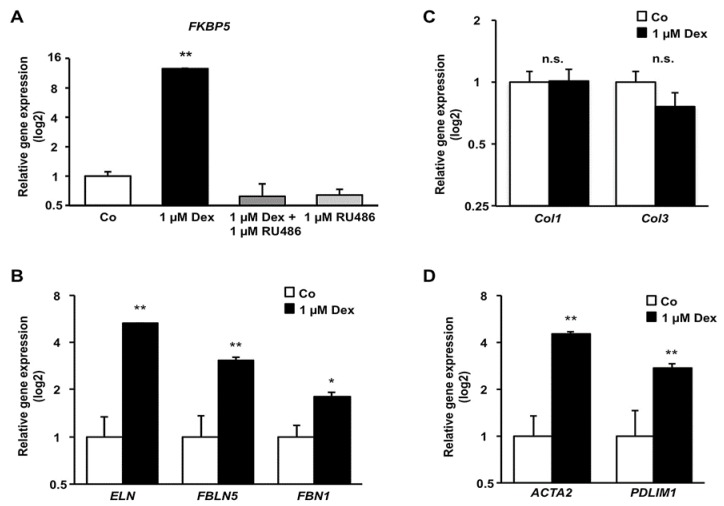
Dex treatment of HTPCs rapidly increases *FKBP5* mRNA, a known target of GR signaling, and affects mRNA levels of characteristic peritubular ECM markers, as well as cytoskeleton-associated genes. (**A**) Cultured HTPCs stimulated with Dex (1 µM) in the absence or presence of RU486 (1 µM), respond with a highly significantly increase (*p* < 0.01) in *FKBP5* mRNA levels after 6 h compared to control. RU486 completely blocks this effect (*p* < 0.01) and does not alter *FKBP5* mRNA expression within 6 h. (**B**) After Dex treatment of HTPCs for 24 h, the mRNAs of the elastic fiber components *ELN* and *FBLN5* are highly significantly (*p* < 0.01) increased. A slightly smaller (*p* < 0.05) increase is observed for *FBN1* mRNA. (**C**) The transcript concentration of the collagen fibers *Col1* and *Col3* do not change (*p* > 0.05) after application of Dex, while (**D**) the mRNAs of cytoskeleton markers *ACTA2* and *PDLIM1* (*n* = 8) are enriched (*p* < 0.01) after stimulation of HTPCs with Dex. Data are means ± SEM after 6 (**A**), and 24 h (**B**–**D**), normalized to control conditions. Asterisks denote statistical significance, * *p* < 0.05, ** *p* < 0.01.

To assess a transcriptionally active and thus functional GR in HTPCs, cells were treated with Dex (1 µM) for 6 h. Then FK506 binding protein 5 (*FKBP5*), a known target of GR signaling, was quantified by qPCR. As shown in Figure 4A, this gene responded to a 6 h Dex treatment with a more than 12-fold increase of mRNA (*p* < 0.01) compared to control. This effect was mediated through GR activation since it was completely blocked (*p* < 0.01) by the simultaneous addition of 1 µM RU486. In contrast, RU486 alone did not alter *FKBP5* mRNA levels after 6 h. This confirms an active GR involved in steroid-responsive pathways in HTPCs. 

### 3.4. Effect of Dex on mRNA Level of Components of the Cytoskeleton and Extracellular Matrix

As shown in Figure 4B, 1 µM Dex produced a more than 5-fold mRNA increase (*p* < 0.01) in the extracellular matrix component *ELN*, and a 3-fold upregulation (*p* < 0.01) of fibulin 5 (*FBLN5*) transcripts, which is essential for elastic fiber formation, after 24 h compared to control cells. A slightly smaller but still significant (*p* < 0.05) stimulatory effect was also observed on fibrillin 1 (*FBN1*) mRNA, inevitable for elastic fiber interaction. In contrast to that, HTPCs did not change their mRNA content for the collagens *Col1* and *Col3* significantly (*p* > 0.05) in response to Dex compared to control (Figure 4C). Dex-treated HTPCs upregulated the mRNA of the cytoskeleton elements *ACTA2* and *PDLIM1* (PDZ and LIM domain protein 1), which associates with actin fibers highly significantly (*p* < 0.01) after 24 h (Figure 4D).

### 3.5. Dex Caused a Rapid Change in Cellular Morphology and an Increase in Stress Fibers

**Figure 5:**
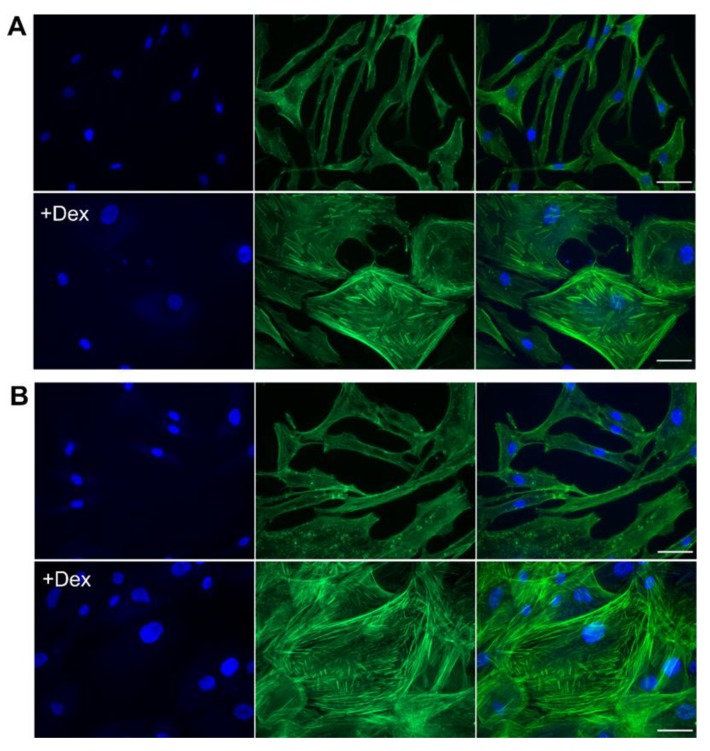
Dex treatment induces an increase in actin stress fibers and a change in cell morphology. (**A**,**B**) Fluorescence microscopy of control HTPCs and cells treated with 1 µM Dex after 24 (**A**) and 72 h (**B**). Upper panels show untreated HTPCs; lower panels Dex-treated HTPCs. Filamentous actin was visualized by staining with Atto 488-phalloidin (middle), and DNA with DAPI (left). Merged images are shown on the right. Scale bars = 50 µm.

The application of 1 µM Dex to HTPCs caused a striking change in cellular size and morphology that became obvious in most cells after 24 h (Figure 5A, lower panel) and were even more pronounced after 72 h of cultivation (Figure 5B, lower panel). In detail, while under basal conditions (untreated), HTPCs were characterized by a slim, spindle-like shape (Figure 5A,B, upper panels, and Figures S1 and S2, upper panel); the application of Dex for 72 h not only induced a significant (*p* < 0.05) increase in cell size (Figure S1A,B), but also caused a massive increase in actin stress fibers and altered cellular morphology (Figure 5B, lower panel and Figure S2, lower panel; a semi-quantitative analysis of filamentous actin structures is shown in Figure S3). In contrast, Dex neither influenced cell number after 72 h (Figure S1C) nor affected cell viability of HTPCs significantly (*p* > 0.05) after 24 h as shown by an ATP-assay (Figure S1D).

### 3.6. Dex Induced Increase of Contractility

**Figure 6:**
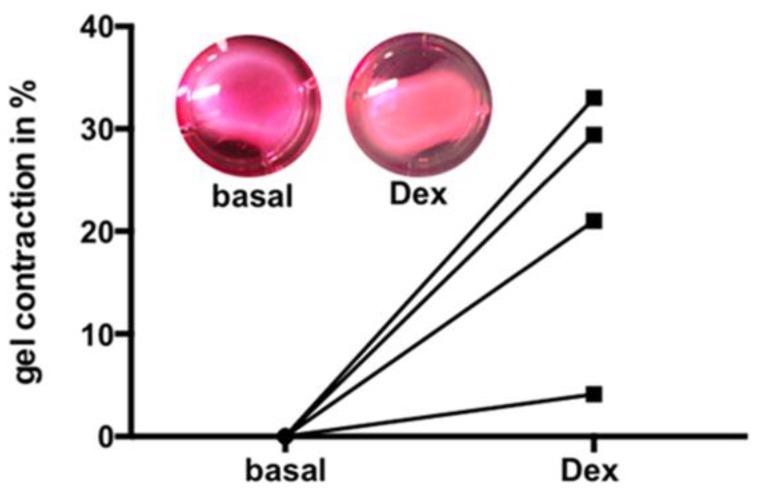
Dex increases contractility in HTPCs. Gel contraction assay indicates the contraction of HTPCs induced by treatment with 1 µM Dex. Decreased gel sizes after stimulation is shown as a percentage of changes of matrix after normalization to solvent control group. Cells from *n* = 4 patients were used. Representative examples are depicted.

To examine the consequences of Dex on contractile characteristics of HTPCs, collagen gel contraction assays were performed, and showed that treatment with 1 µM Dex (72 h) resulted in an increased ability of HTPCs to contract spontaneously. The degree of constriction varied between cells derived from four different donors (Figure 6).

### 3.7. Electric Cell Substrate Impedance Sensing (ECIS) Measurements to Quantify Cell Behavior

**Figure 7:**
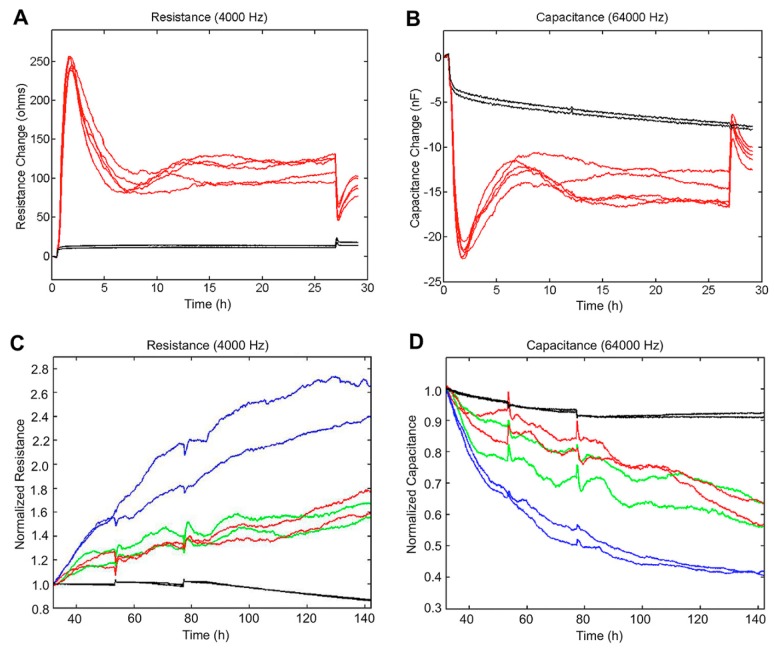
ECIS measurements with HTPCs and effects of treatment with Dex (**A**,**B**). Characterization of HTPC growth after seeding of HTPCs onto ECIS electrodes (0 h). Barrier resistance representing the tightness of cell-to-cell contacts, was measured at 4000 Hz, after preliminary experiments to optimize resistance measurements (**A**) and capacitance at 64,000 Hz (**B**). Black lines correspond to the cell-free controls. After 27 h, 1 µM Dex was added. EtOH-treated and untreated cells served as controls. (**C**) Resistance at 4000 Hz after application of Dex compared to control cells normalized at t = 32 h. Red curves correspond to the untreated control cells, green curves to EtOH-control cells, and blue curves to Dex-treated cells. (**D**) Capacitance at 64,000 Hz in Dex-treated and control HTPCs normalized at t = 32 h. The black lines in panels C–D correspond to the cell-free controls. In parallel experiments performed with a second HTPC line, similar results were obtained.

In order to quantify the cellular response to Dex treatment, ECIS measurements were performed (Figure 7). In the first experimental step, HTPC cultures grown on ECIS electrodes were validated in normal proliferative conditions (Figure 7A,B). In the second step, HTPCs treated with Dex in comparison to untreated or EtOH-treated control cells were measured. Dex-treated HTPCs showed a strong increase in resistance, indicating proliferation or increased cell–cell contact formation (Figure 7C), whereas the membrane capacitance of Dex-treated cells decreased (Figure 7D). The decrease in capacitance of Dex-treated cells indicates a larger coverage area on electrodes, and is in accordance with the enlarged cellular morphology observed by immunofluorescence.

## 4. Discussion

GC are crucial steroid hormones, which are involved in body homeostasis and in stress reactions [23]. Furthermore, several drugs, including Dex, act at the GR and are widely used in men for medical reasons. A previous study documented the GR in several cells of the human testis. While some Leydig cells, endothelial cells, smooth muscle cells of blood vessels, a subset of spermatogonia, and possibly Sertoli cells were immuno-positive, the study showed that GR is predominantly expressed by peritubular cells [4]. The mentioned results were confirmed by our study. They reveal multiple targets and thus implicate different actions of GC at the level of the male gonad. So far, however, very little is known about the consequences of testicular GR activation. Clearly, in humans, such actions are difficult to examine and adequate translational models are required. We reasoned that studies employing both nonhuman primate (NHP) testes and studies in isolated HTPCs may help to elucidate GR-mediated actions.

In adult human testes and in NHP testes, we confirmed the previously described localizations of GR, specifically the expression by peritubular cells. Further, in NHP testes samples, GR-immunoreactive peritubular cells were detected only after the onset of puberty. How GR-expression is developmentally regulated in the testis remains to be studied. Yet, the results are in line with the ones reported in human fetal testes samples [4] and collectively suggest that due to absent GR, GC do not play a role in the regulation of peritubular cell functions before puberty.

Peritubular cells of the adult testis can contract and relax. Thereby, waves of the wall of seminiferous tubules are generated, which are considered crucial for the intra-testicular transport of sperm. Peritubular cells of adult men contain smooth muscle actin (ACTA2) and produce extracellular matrix components. For example, the extracellular matrix of the peritubular wall contains collagens and ELN (see images in the Human Protein Atlas; [11]). Both contractility of peritubular cells and the overall elastic properties of the wall may conceivably contribute to sperm transport, albeit to our knowledge such actions have not been examined. Elastic fibers were reported to be sparse or absent in the peritubular wall of young men with hypogonadotropic hypogonadism [24]. This indicates a link to sexual maturity. In addition, glucocorticoid response elements were reported in the human *ELN* gene, and GC enhanced ELN synthesis in a mouse model [25].

We found that nascent peritubular cells not only lack GR, but that also ACTA2, and ELN were undetectable in the wall compartment in prepubertal, sexual immature testes. Col1 in the wall compartment was detected before and after puberty. The results suggest that GR signaling in adult peritubular cells may be associated with the regulation of the differentiation state of peritubular cells, namely contractile properties, as well as ELN production.

A puberty-associated induction of ACTA2 and hence acquisition of contractile abilities was previously described in peritubular cells of NHP [14], but changes in ELN or a link to GR-signaling were unknown, to our knowledge. A regulation of ACTA2 by androgens, via AR, was recently shown in HTPCs [26]. The results obtained now raise the possibility that besides AR, also GR may be involved in the regulation of the contractile, smooth muscle-like phenotype of HTPCs and in the elastic properties of the peritubular wall.

To explore such a possibility, we tested HTPCs, after confirming that isolated HTPCs express functional GR. This was indicated by the rapid nuclear translocation of the receptor upon stimulation. Further, RU486, an antagonist at the GR prevented *FKBP5* induction, typically associated with active GR [27,28].

The results pinpoint GRα, i.e., the biologically active splice variant of the GR, which was also readily detected in HTPCs, among others by Western blot. A previous study employed also Western blotting and reported the full length GRα in whole human testes homogenates [1]. In our study, the same antibody was used for both Western blot and immunohistochemistry, and hence, we conclude that GRα is present also in situ. Clearly, we cannot rule out that low levels of other variants, which are described in humans, including the GRβ variant, may also be expressed [29]. Future studies will also have to address the single nucleotide NR3C1 polymorphisms described [1,4].

Using the cellular model of HTPCs, we found that GR activation by Dex significantly increased *ACTA2*, *ELN* and associated protein transcript levels. They include *PDLIM*, *FBLN5* and *FBN1*. In contrast, *Col1/3* were not affected by Dex. Dex treatment of HTPCs also had striking consequences on cell size and form, as well as on the actin-cytoskeleton. Thus, HTPCs lost the typical slim spindle-like shape, and phalloidin-labeling revealed a massive increase in actin stress fibers.

Previously, cellular hypertrophy and increases in muscle markers as a consequence of Dex were described in studies of cardiomyocytes [30], indicating that GR signaling may be involved in the regulation of other muscle types, as well. In the male murine heart, GCs indeed play a crucial role in maintaining normal cardiac functions [31], a possibility that now remains to be tested in the male gonad. In trabecular meshwork cells, cytoskeletal changes upon Dex are also well documented, and include e.g., PDLIM1 [32].

To examine consequences of Dex on contractile abilities of HTPCs, we performed collagen gel contraction assays. As expected from patient-derived cells [26], variations in the degree of the response became apparent. Yet all experiments performed with cells from four men showed that Dex resulted in an increased contraction of gel matrices and confirm an increase in contractile properties.

Results of ECIS measurements, namely the strong increase in resistance, further imply increased cell–cell contact formation, as increased cell proliferation was not detected. The lower membrane capacitance of Dex-treated cells is in line with increases in cells size and indicates a larger coverage area on electrodes. The results are in agreement with the observed morphological changes upon Dex treatment.

## 5. Conclusions

The study shows that peritubular cells of the adult human testis are a target of GC. These hormones are likely involved in the maintenance of the smooth muscle-like cellular phenotype of peritubular cells and ELN production. Provided that the results can be transferred to the in vivo situation, GR activation may be involved in the post-pubertal maintenance of the contractile abilities of HTPCs and overall elastic properties of the peritubular wall, which both appear to be relevant to sperm transport. Other roles of GR activation in peritubular cells and in the testis remain to be studied.

## Supplementary Materials

The following are available online at https://www.mdpi.com/2077-0383/9/4/961/s1, Figure S1. (A) Effect of a 72 h incubation period with Dex (1 µM) compared to control (Basal, medium) on cell morphology (A, two representative pictures are shown), (B) cell diameter, (C) proliferation of HTPCs. (D) Cell viability of HTPCs as determined by an ATP assay after a 24 h incubation period with Dex (1 µM); *n* = 3, with quintuplicates in each experimental group. Paired *t*-test, values are the mean ± SEM. Asterisk denotes statistical significance, * *p* < 0.05; n.s.: not significant; Figure S2. Fluorescence microscopy of control HTPCs (upper panel), and HTPCs derived from another patient line than the cells shown in Figure 5 treated with Dex (1 µM; lower panel) for 72 h. Filamentous actin was visualized by staining with Atto 488-phalloidin (middle), and DNA with DAPI (left). Merged images are shown on the right. Scale bars = 50 µm; Figure S3. Semiquantiative analysis filamentous actin structures in HTPCs (A,B). Control HTPCs (A) were cultivated in parallel to cells treated with Dex (1 µM) for 24 h (B). The images were recorded with identical microscope settings, and two examples are shown for control (A), and Dex-treated cells (B). Left images depict the overlay of the green (filamentous actin visualized by staining with Atto 488-phalloidin) and blue channels (nuclei visualized by staining of DNA with DAPI). Images on the right indicate the intensity profiles depicted in the 16-color mode (Fiji) of grey values of the green (actin) channel. Scale bars = 50 µm; Table S1. Forward (For) and reverse (Rev) primer sequences (5′-3′) employed in real time PCR, amplicon size, annealing temperature (A-Temp.), and source (accession number); n.a.: not applied in real time PCR.

## Further Information

**Human Protein Atlas:**https://www.proteinatlas.org; **3web software**: http://primer3.wi.mit.edu.
