# Supplementary material for: The Glucocorticoid Receptor NR3C1 in Testicular Peritubular Cells is Developmentally Regulated and Linked to the Smooth Muscle-Like Cellular Phenotype
Source: J Clin Med. 2020 Mar 31;9(4):961. doi: 10.3390/jcm9040961 (PMC7230580; doi:10.3390/jcm9040961)
Supplement: Supplementary file 1 [file jcm-09-00961-s001.pdf]

Journal of
Clinical Medicine

MDPI

Supplementary Materials

The Glucocorticoid Receptor NR3C1 in Testicular Peritubular Cells is Developmentally Regulated and Linked to the Smooth Muscle-like Cellular Phenotype

Harald Welter[1*], Carola Herrmann[1], Nils Dellweg[1], Annika Missel[1], Christiane Thanisch[2], Henryk F. Urbanski[3], Frank-Michael Köhn[4], J. Ullrich Schwarzer[5], Annette Müller-Taubenberger[1] and Artur Mayerhofer[1*]

Table S1. Forward (For) and reverse (Rev) primer sequences (5′-3′) employed in real time PCR, amplicon size, annealing temperature (A-Temp.), and source (accession number); n.a.: not applied in real time PCR.

Gene Name	Sequence (5′- 3′)	Amplicon size (bp)	A-Temp. (°C)	Source (Accession Number)
RPL19	For AGGCACATGGGCATAGGTAA<br>Rev CCATGAGAATCCGCTTGTTT	199	59	NM_000981.3
GRα	For AGCCATTGTCAAGAGGGAAG<br>Rev AGCAATAGTTAAGGAGATTTTCAACC	113	n.a.	NM_001018077
GRβ	For AGCCATTGTCAAGAGGGAAG<br>Rev TTTCTGGTTTTAACCACATAACATTT	110	n.a.	NM_001020825
GR(*NR3C1*)	For GAAGGAAACTCCAGCCAGAA<br>Rev GATGATTTCAGCTAACATCT	159	60	NM_000176.3
ACTA2	For ACCCAGTGTGGAGCAGCCC<br>Rev TTGTCACACACCAAGGCAGT	110	62	GU143396.1
ELN	For CACTGGGGTATCCCATCAAG<br>Rev CCATAGCCATAGGGCAGTTT	85	60	NM_000501
Col1	For CACACGTCTCGGTCATGGTA<br>Rev AAGAGGAAGGCCAAGTCGAG	91	58	NM_000088.3
Col3	For GGTGGTTTTCAGTTTAGCTACGG<br>Rev TGATGTTCTGGGAAGCTCGG	106	60	NM_000090.3
FBLN5	For CAATTTACAAGGGGGCTTCA<br>Rev GGGTTCTCAGCAGGACACAT	99	59	NM_006329.3
PDLIM1	For TCAAAGGCTGCACAGACAAC<br>Rev TATGGATGACGCTTCCCTTC	97	60	NM_002615
FKBP5	For GCATTATCCGGAGAACCAAA<br>Rev GCCACATCTCTGCAGTCAAA	121	59	NM_004117.3
FBN1	For ATGTGAATGCTTCCCTGGAC<br>Rev GGCCTCTCTTGTATCCACCA	98	59	NM_000138.5

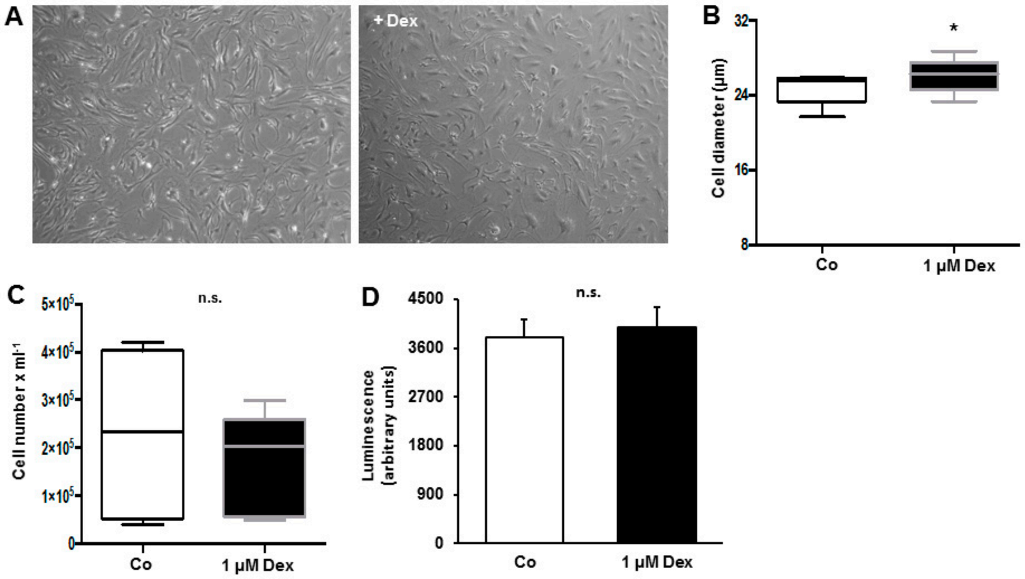

Figure S1. (A) Effect of a 72 hours incubation period with Dex (1 µM) compared to control (Basal, medium) on cell morphology (A, two representative pictures are shown), (B) cell diameter, (C) proliferation of HTPCs. (D) Cell viability of HTPCs as determined by an ATP assay after a 24 hours incubation period with Dex (1 µM); n = 3, with quintuplicates in each experimental group. Paired t-test, values are the mean ± SEM. Asterisk denotes statistical significance, $*p < 0.05$; n.s.: not significant;.

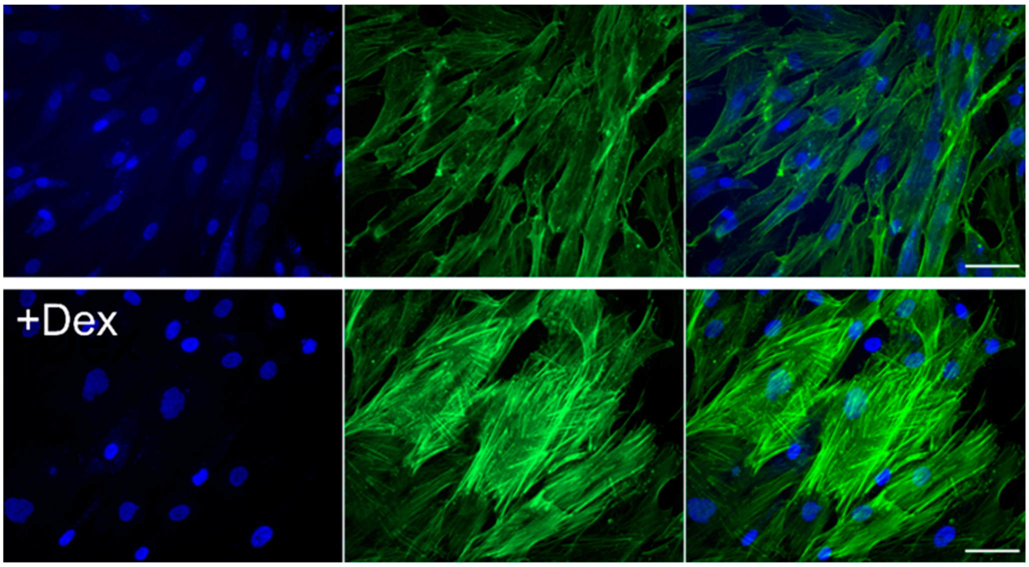

Figure S2. Fluorescence microscopy of control HTPCs (upper panel), and HTPCs derived from another patient line than the cells shown in Figure 5 treated with Dex (1 µM; lower panel) for 72 hours. Filamentous actin was visualized by staining with Atto 488-phalloidin (middle), and DNA with DAPI (left). Merged images are shown on the right. Scale bars = 50 µm;

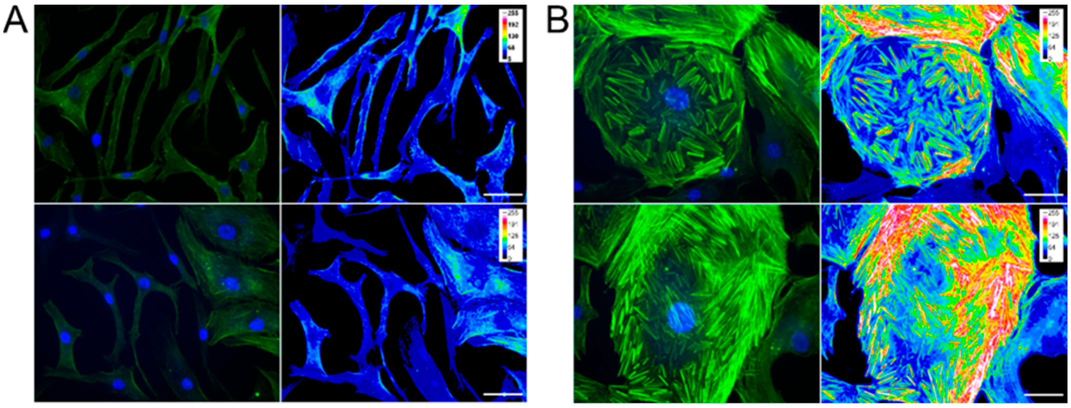

.

Figure S3. Semiquantiative analysis filamentous actin structures in HTPCs (A,B). Control HTPCs (A) were cultivated in parallel to cells treated with Dex (1 μM) for 24 hours (B). The images were recorded with identical microscope settings, and two examples are shown for control (A), and Dex-treated cells (B). Left images depict the overlay of the green (filamentous actin visualized by staining with Atto 488-phalloidin) and blue channels (nuclei visualized by staining of DNA with DAPI). Images on the right indicate the intensity profiles depicted in the 16-color mode (Fiji) of grey values of the green (actin) channel. Scale bars = 50 μm;.
